# Continuously adjustable hollow beam for ultrafast laser fabrication of size-controllable nanoparticles

**DOI:** 10.1515/nanoph-2024-0690

**Published:** 2025-03-27

**Authors:** Zhi Wang, Peng Yi, Andong Wang, Taoyong Li, Wentao Chen, Xiaolin Qi, Xiaowei Li

**Affiliations:** School of Mechanical Engineering, 47833 Beijing Institute of Technology, No. 5 Zhonggguancun South Street, Haidian Beijing, Beijing 100081, China; Laser Micro/Nano Fabrication Laboratory, School of Mechanical Engineering, 47833 Beijing Institute of Technology, Beijing 100081, China

**Keywords:** spatial shaping, femtosecond laser, controllably sized nanoparticles, hollow beam

## Abstract

The focused vortex beam generates a hollow beam, which has been widely used for size-controlled nanoparticle formation on various materials. However, the size variation of the vortex beam is limited by the integral order of the 2π phase wrap, while the waste is caused by the large side lobe around the center. In this study, we propose a method for hollow beam generation by splitting a femtosecond laser and imparting opposite phases to the outer annular region and the central Gaussian region. After focusing, these two regions overlap at the focal spot, resulting in a hollow beam due to phase cancellation. By modulating the relative dimensions of these two regions, the hollow center can be continuously varied. When such a hollow beam is used for surface processing, the thermal capillary effect facilitates the convergence of the molten material toward the center, ultimately leading to the formation of nanoparticles. This ability to control size allows precise control of nanoparticle size with a diameter range from 140 nm to 940 nm. This method holds great promise for guiding research into nanoparticle properties that are influenced by size effects.

## 1 Introduction

In recent years, nanoparticles have garnered significant attention due to their intriguing properties in the fields of optical [1], [2], [3], electrical [4], [5], [6], and catalytic [7], [8], [9] domains. These properties are intricately linked to the shape, size, and uniformity of the nanostructure. The surface lattice resonance (SLR) [10], [11], [12] of metal nanoparticle arrays enables the investigation of sensing performance in mixed plasma-photon modes. It also plays a role in light–matter interactions, including lasing [13], [14], [15], [16] and strong coupling [17], [18], [19]. Extensive research has been conducted on the flexible adjustment of size, shape, material, lattice spacing, and array arrangement period [20] to influence the wavelength range and spectral width of reflected light intensity. To systematically explore the diverse characteristics of nanoparticles, it has become a critical research focus [21] to develop a method for the controlled synthesis of nanoparticles with well-defined morphology. Therefore, there is an urgent need for a straightforward, cost-effective approach that enables efficient size-controlled synthesis of nanoparticles.

Femtosecond laser [22], [23], [24], [25], [26], [27] direct writing represents an ideal approach to address the aforementioned challenges, owing to its characteristics of mask-free operation, absence of a vacuum processing environment, and elimination of chemical treatments. Previously, many works have focused on vortex beams to generate hollow beam as a tool for nanoparticle generation. Due to the easy generation of the vortex beam, this method has been widely used on different materials including silicon [28], metal film [29], [30], tantalum [31], and graphene [32]. Nevertheless, traditional approaches for creating hollow beams via focusing a vortex beam exhibit limited flexibility in controlling beam hole diameters (Figure S1). The vortex beam obtained through the application of vortex phase shaping exhibits a spiral distribution as it propagates in different directions and distances, while maintaining its distinctive phase distribution: $\phi \left(r,\theta ,z\right)=-l\theta +kz$ , where the integer *l* called the topological charge (TC), which determines the spiral wavefront distribution of the vortex beam. On condition that the vortex phase change is an integer order of 2π, the focusing spot will show a closed hollow beam. However, this limits the continuous size change of the hollow center, thereby complicating the precise adjustment of nanoparticle sizes. Another drawback of the vortex hollow beam is the large material waste during the nanoparticle fabrication. Because the proportion of the hollow center of the vortex beam is relatively small, this property leads to significant sidelobe damage in the processing of vortex beams on nanoparticles, resulting in low particle preparation density and an unstable distribution period [31], [32], [33], [34], [35].

**Figure 1:**
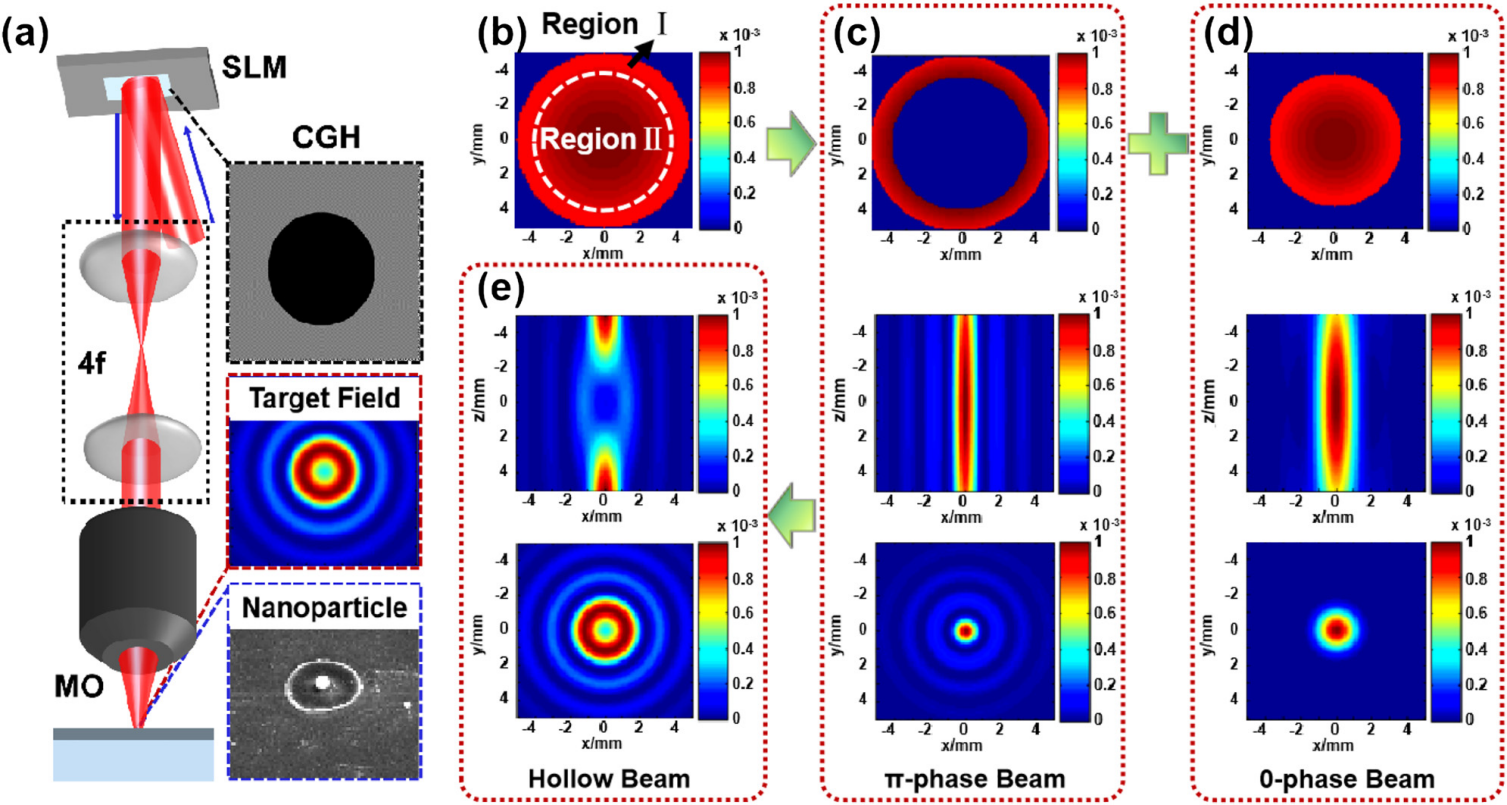
The schematic representation illustrating nanoparticle processing methodologies (a) and hollow beam designs (b–e), encompassing the traditional Gaussian spot (b), π-phase ring spot (c), 0-phase central spot (d), and the resultant combined hollow beam (e).

In this study, we present a novel approach for generating hollow beams with adjustable dimensions. A spatial light modulator (SLM) is employed to partition the femtosecond laser input into a central region and an annular edge region (Figure 1(a)). The surrounding annular light field is assigned a 0 phase, while the central light field is imparted with a π phase. Upon focusing through a lens, the opposing phases of the light fields result in cancellation at the focal point. By varying the diameter of the π phase region, we can achieve flexible control over the size of the hollow beam’s center. Subsequently, this hollow beam is directed onto material surfaces; as it focuses, it induces melting flow within its central area that facilitates precise adjustment of nanoparticle sizes at that location. Furthermore, this method demonstrates extensive material compatibility and enables stable generation of size-tunable nanoparticles on various thicknesses of metallic thin films and bulk materials, allowing for flexible adjustments in particle sizes ranging from 140 nm to 940 nm. The performance of this fabrication method is compared with that of the focused vortex beam, demonstrating its high flexibility and low material waste.

## 2 Results and discussion

Figure 1 illustrates the concept of beam shaping and nanoparticle processing. The conventional intensity distribution of femtosecond lasers is characterized by a Gaussian profile (Figure 1(b)), with its thereby complicating efforts to further minimize the spatial dimensions of the focused spot. Consequently, this study delineates the illuminated area into region I (the outer region beyond the white dashed line) and region II (the inner region within the white dashed line). Focusing precision constrained by the optical diffraction limit, for region I, leveraging off-axis illumination (OAI) as an important technique to enhance imaging resolution allows us to express traditional laser focusing resolution as follows [36]:

(1) $$CD_{1}=\frac{\lambda }{2NA\left(1+\sigma \right)}$$

where *σ* = *n*_*i*_ sin*φ*/*NA* is a partial coherence factor, *n*_*i*_ is the refractive index of sample, and *φ* is the angle between the edge light and the main light (Figure S2(a)).

In the context of employing a ring-shaped holographic focusing technique, this method is equivalent to focusing light solely within the range of *ε*N.A. − N.A., in contrast to conventional laser focusing (where *ε* denotes the ratio of the inner and outer diameters of the ring-shaped holographic field) (Figure S2(b)). Consequently, the focusing resolution can be articulated as follows [21]:

(2) $$CD_{2}=\frac{\lambda }{2NA\left(1+\sigma \right)+\Theta }$$

A comparison of Equations (1) and (2) indicates that the introduction of the tilt factor in off-axis illumination (OAI) significantly enhances focusing resolution, thereby achieving a reduced focused spot size (Figure 1(c)) relative to the light field in region II.

Utilizing the SLM to impart π-phase and 0-phase to the light fields in region I and II, respectively, induces a phase shift between the two components. After converging by an objective lens, the two parts are focused at the same positon, leading to the cancellation of light intensity at the overlapping region due to the opposite phase value, thereby achieving a hollow beam (Figure 1(c)). The shaped beam is subsequently focused onto the material surface (Figure 1(a)) for the fabrication of nanoparticles on the sample surface.

The intensity profile of the combined beam after focusing is greater than that of the individually focused beams in region I and region II. This phenomenon arises because the phase at the center of the beam is inverted during the focusing process, which leads to cancellation. The unoffset portion of region II, unaffected by the region I beam, is retained as the new energy focal point. Due to its diminished strength compared to before, a higher power density is necessary for effective material removal, resulting in a larger spot size that achieves efficient material elimination. This effect facilitates material ablation of the surrounding material and benefits compressing the melted material toward the central region of the light field.

**Figure 2:**
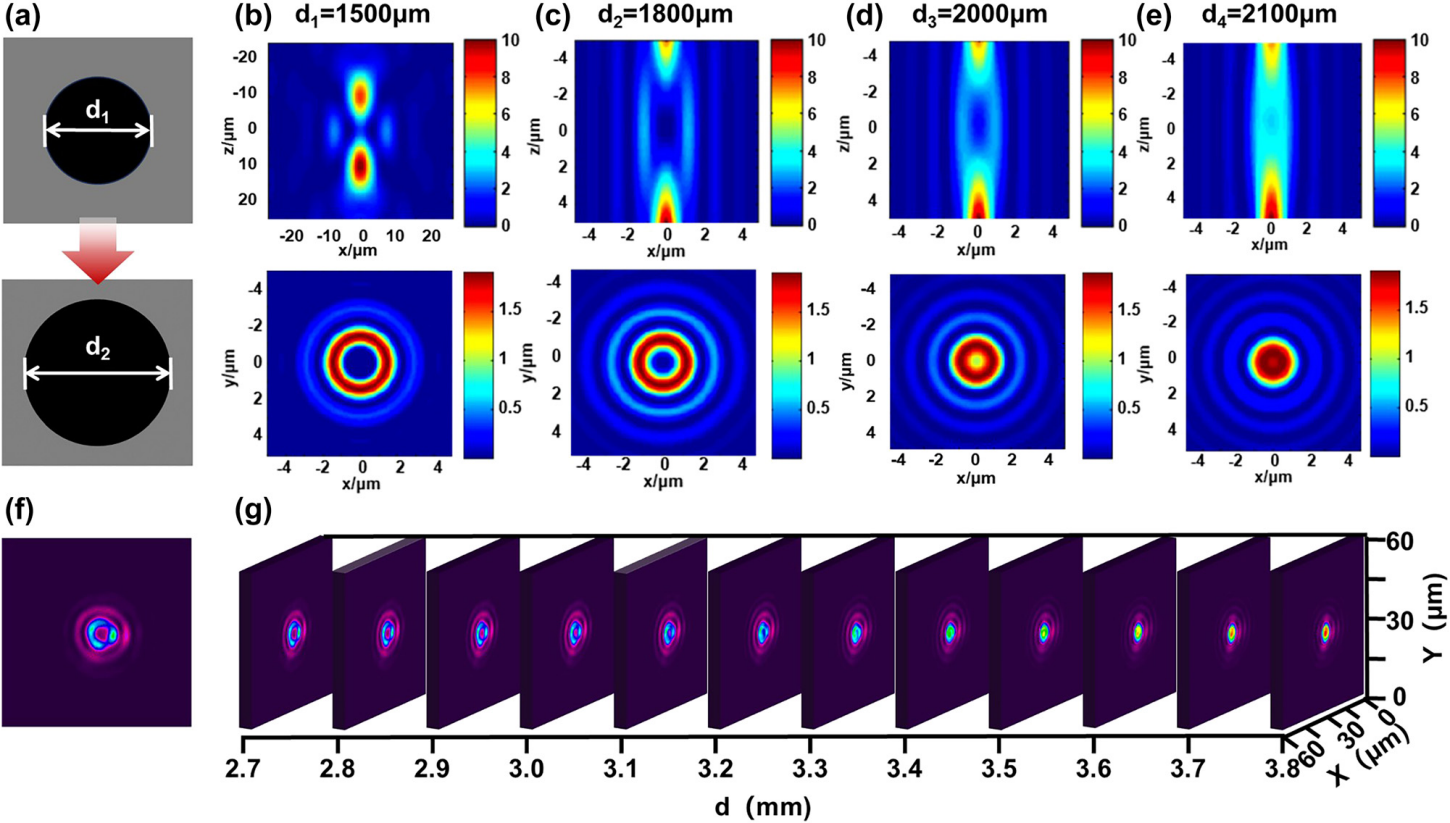
Schematic of illustrating the adjustment of the central size of the hollow beam (a), with central diameters of 1,500 μm, 1,800 μm, 2,000 μm, and 2,100 μm; simulated axial and lateral intensity distributions of the hollow beam in MATLAB (b–e) corresponding to a central diameter of 1,800 μm; as well as measured intensity at the focal point (f) and the measured results of focal light field intensity distribution with *d* varying from 2.7 mm to 3.8 mm (g).

By adjusting the relative dimensions of region I and II (Figure 2(a)), the size ratio of the focused spots in these two regions can be modulated, thereby enabling flexible control over the diameter of the hollow beam void. The axial and lateral light fields were computed and analyzed using MATLAB simulations (incident wavelength: 800 nm, incident spot size *D* = 10 mm, objective *NA* = 0.45).

As illustrated in Figure 2(b)–(e), gradually increasing the diameter of the central region from 1,500 μm to 2,100 μm while concurrently reducing the size of the annular light field region (region I) results in a diminished light intensity after focusing. This leads to a reduction in the area where cancellation occurs at the center, causing a systematic decrease in light intensity at that location. When *d* = *d*_1_ = 1,500 μm, the central void diameter is ≈3.2 μm; as *d* increases to 2,100 μm, this diameter reduces to ≈800 nm, facilitating flexible regulation of hollow beam void dimensions. Furthermore, for *d* ≥ 2,000 μm, a void region measuring about 4 μm exists within the axial light intensity distribution, effectively mitigating disturbances caused by defocus and oscillation during femtosecond laser processing. As *d* continues to decrease, it results in an imbalance in the intensity ratio between region I and region II. This imbalance leads to the formation of a concentric secondary focal point at the center of the hollow beam, which subsequently affects the effective preparation of nanoparticles (Figure S4).

Additionally, a test configuration utilizing a beam quality meter was established to evaluate the actual light field of the hollow beam generated by the designed system, as illustrated in Figure S3. The results presented in Figure 2(f) and (g) demonstrate that the hollow beam light field is effectively shaped at the focal position (Figure 2(f)). Moreover, the characterization of the light field at the focal point in the case of continuous variation of *d* demonstrates a consistent change trend with the simulation analysis process. As *d* gradually increases, the intensity of region I carrying π-phase diminishes gradually, preventing effective phase cancellation and resulting in a gradual transformation of spot shape into a Gaussian beam, thereby ensuring fabrication reliability.

**Figure 3:**
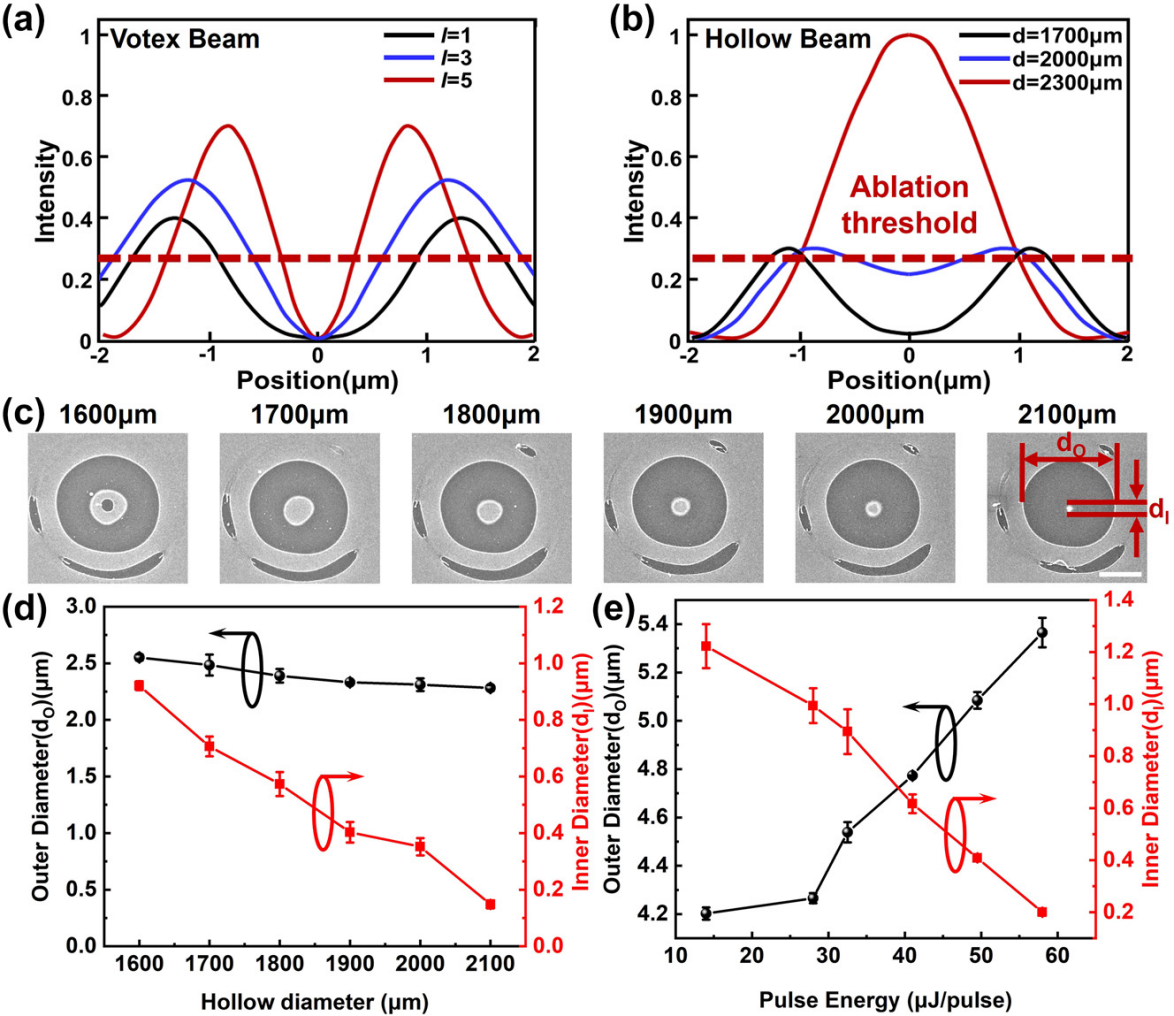
Simulation of the intensity distribution of vortex beam with *l* = 1, 3, 5 (a) and hollow beam with *d* = 1,700 μm, 2,000 μm, 2,300 μm. (b) The red dashed line in the figure represents the ablation threshold of 20 nm Au film. Characterization of the 20 nm Au film surface via scan electron microscopy (SEM) following irradiation with light fields of varying region II diameter (b) (scale bar: 2 μm), along with the corresponding variations in the outer and inner diameters of the annular region under different region II diameters (d) and pulse energy (e).

The application of the aforementioned shaped hollow beam in the processing of membrane materials facilitates the controlled synthesis of nanoparticles. In contrast to vortex beam (Figure 3(a)), hollow beams enable flexible modulation of light intensity in the central region while maintaining a nearly constant focal spot size (Figure 3(b)). During processing with a vortex beam, regulation of weak light intensity within the central area is achieved by adjusting the helical degree of its phase structure. As this helical degree increases, both the size of the central region and that of the focused spot significantly expand, leading to excessive side lobe ablation during fabrication (Figure 3(a)) (Figure S5). The hollow beam allows for adaptable control over the dimensions of the weak light region through relative adjustments between region I and II. Importantly, while center light intensity does not reach zero, it exhibits a continuous variation corresponding to changes in region I’s size. Given a specific material system where burnout thresholds remain fixed, one can not only regulate dimensions within this weak light zone but also modulate its intensity relative to these thresholds – thereby influencing dynamics such as volume and flow velocity within molten centers – ultimately allowing for precise control over both side lobe damage areas and nanoparticle sizes.

Subsequently, the reshaped hollow beam was directed onto the surface of a 20 nm Au film on glass substrate (Figure 3(c)) using a 20× objective lens with *NA* = 0.45 and pulse energy *F* = 14 μJ/pulse. The unique intensity distribution of the hollow beam creates an intensity void at its center, while the annular region with higher light intensity facilitates effective material removal. The gold film melts into liquid and is drawn toward the central void through thermal capillary action, enabling nanoparticle fabrication. As the size of region II increases gradually, the residual material size at the center of the void diminishes correspondingly. This reduction can be attributed to two primary factors: First, a decrease in the size of the intensity void at the focal point removes more surrounding material; Second, as π-phase field intensity decreases, more laser energy becomes necessary for material removal, leading to an accumulation of excess laser flux that destabilizes molten gold and ultimately results in its ejection from the substrate – thereby further reducing gold content available for nanoparticle synthesis.

Moreover, when the diameter of the central particle in region II is 1,600 μm, a void is observed at the center. This phenomenon arises from the significant disparity in light intensity ratios between region I and II, which results in a larger focused spot in region I after focusing, accompanied by residual laser flux that ultimately leads to secondary ablation of the particle. Characterization results indicate that the dimensionally adjustable hollow beam can effectively facilitate flexible regulation of nanoparticle size.

Through statistical analysis of the outer diameter (*d*_O_) and inner diameter (*d*_I_) under varying diameters of region II (Figure 3(d)) and different incident laser fluence (Figure 3(e)), the results indicate that adjusting the diameter of region II allows for *d*_O_ to remain nearly constant while enabling flexible adjustments to *d*_I_, thereby effectively controlling feature size. In contrast, modifications to the laser flux are likely to result in excessive values, leading to instability in both *d*_O_ and *d*_I_, which hampers effective control over feature dimensions. The comparison between these two approaches further underscores the efficacy of regulating feature size through manipulation of the region II diameter.

The hollow beam exhibits fewer sidelobe damage compared with the traditional vortex phase shaping vortex beam machining results. Adjusting the helicity of the vortex phase-generated vortex beam is necessary to modify the size of its central hole, which in turn increases the spot size and eventually leads to severe sidelobe damages. In contrast, by adjusting the relative intensity of region I and region II instead of altering the zero size of center light intensity, the hollow beam can flexibly regulate light intensity at its focal point. This enables nanoparticle formation through threshold effects while maintaining a small and stable range for sidelobe damage.

**Figure 4:**
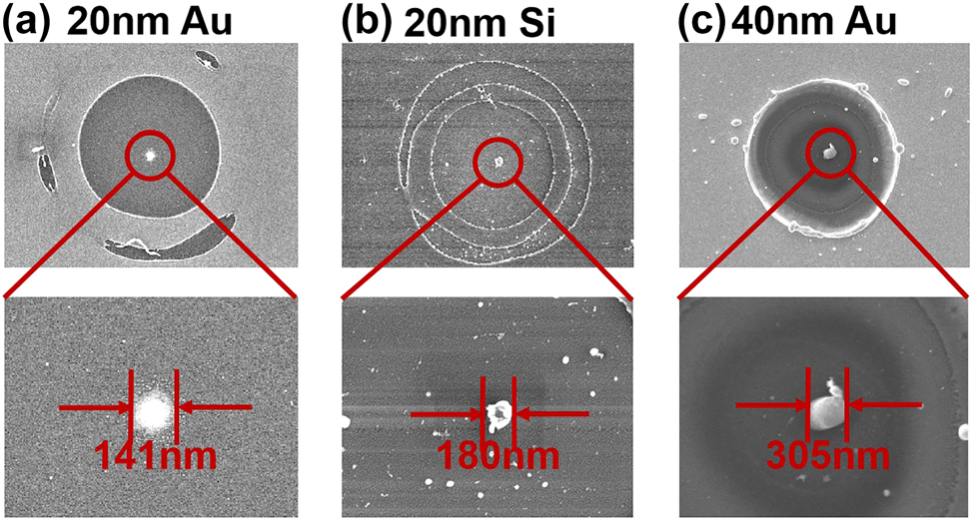
Characterization of the fabrication results for 20 nm Au (a), 20 nm Si (b), and 40 nm Au (c) under laser irradiation conditions of *d* = 1,600 μm and *F* = 14 μJ/pulse.

The nanoparticle size processed from the 20 nm gold film can reach as small as 140 nm (Figure 4(a)), which is one-sixth of the incident laser wavelength, thereby surpassing the optical diffraction limit in terms of size precision. Furthermore, this method demonstrates broad applicability across various material systems, including semiconductor thin films (20 nm Si in Figure 4(b)) and gold films of varying thicknesses (40 nm Au in Figure 4(c)). In comparison to 20 nm Au, Si exhibits lower thermal conductivity and greater mechanical strength, resulting in reduced surface melting of Si nanoparticles under hollow beam irradiation and consequently sharper surfaces for these nanoparticles. Additionally, while thicker gold films significantly diminish side lobe effects under identical laser flux conditions, the increased volume of molten Au leads to a notable expansion of both the edge recast layer and central void convergence, ultimately causing a slight increase in nanoparticle size to 305 nm.

**Figure 5:**
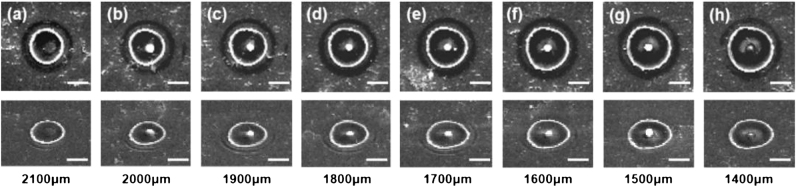
The characterization of the results from laser irradiation of bulk silicon in region II with dimensions *d* = 2,100 μm (a), 2,000 μm (b), 1,900 μm (c), 1,800 μm (d), 1,700 μm (e), 1,600 μm (f), 1,500 μm (g), and 1,400 μm (h) is conducted. (scale bar: 2 μm).

Hollow beams can effectively generate nanoparticles on the surface of bulk materials, as illustrated in Figure 5, exhibiting a trend similar to that observed in thin film materials. When the size of region II is excessively large (*d* = 2,100 μm) (Figure 5(a)), the weak light intensity in region I hampers effective cancellation with the focused light field in region II; consequently, the light intensity at the center of the focused spot remains above the material ablation threshold, leading to nanoparticle disappearance. Conversely, when the size of region II is reduced to below 2,000 μm (Figure 5(b)–(h)), an increase in light intensity within region I and a decrease within region II result in a reduction of light intensity at the center of the focused spot below the material ablation threshold. This generates a hollow beam and facilitates nanoparticle formation, allowing for a minimum nanoparticle size reduction down to 248 nm.

The trend in the processing outcomes of bulk silicon materials closely mirrors those observed in multilayer film materials. The size of nanoparticles derived from bulk silicon decreases as the central region size *d* diminishes. However, compared with multilayer films, nanoparticles obtained from bulk silicon are consistently larger. Even under optimal processing conditions with *d* = 1,400 μm, the nanoparticles from bulk silicon remain larger than those produced from silicon films. This discrepancy can be attributed to the distinct optical transmission processes and material removal volumes during femtosecond laser irradiation of bulk materials versus multilayer films. In multilayer film materials such as silicon films, femtosecond laser processing induces surface plasmon polariton excitation at interfaces. This phenomenon is essentially an electromagnetic surface wave generated by the interference between the plasma produced by oscillating electrons in silicon and the incident electromagnetic field. The theoretical processing precision of this surface wave can reach the optical diffraction limit, enabling the fabrication of nanoparticles with smaller spatial dimensions. Surface plasmons primarily form at the interfaces between silicon and fused silica, as well as between silicon and air. However, since surface plasmon generation requires laser energy near the ablation threshold, both silicon films and bulk silicon materials are processed with pulse energies exceeding this threshold to ensure effective material removal, which inhibits the efficient excitation of surface plasmons at the silicon–air interface. Moreover, bulk silicon experiences deeper ablation craters during femtosecond laser processing, leading to a greater material removal volume and consequently more silicon accumulation at the nanoparticle center, resulting in larger particle sizes. Conversely, the limited volume of damaged material in silicon films results in smaller nanoparticle sizes during processing. Furthermore, during the processing of bulk silicon using hollow beams, the absence of a heterojunction between the semiconductor and the dielectric results in all laser energy being deposited directly into the relatively more vulnerable silicon substrate. This leads to a Gaussian distribution of the bottom morphology (Figure S7). The volumes of both the removed and remelted areas increase, causing an accumulation of excess material at the edges, which consequently limits the quality of nanoparticle preparation.

## 3 Conclusions

In summary, this study decomposes the traditional Gaussian beam into a ring-shaped beam and a central Gaussian beam, imparting a π phase to the ring-shaped beam and a 0 − π phase to the central Gaussian beam using a spatial light modulator. The beams with opposite phases are focused in both space and time at the same point, leading to interference cancellation and enabling the shaping of hollow beams. In contrast to spiral beams, the hollow beams described herein allow for flexible adjustment of the ring-shaped beam size, thereby facilitating control over the dimensions of the hollow core while maintaining a constant external diameter. The nanoparticle size can be regulated flexibly within a range from 140 nm to 940 nm, with successful verification across various material systems, providing an effective approach for precise and controllable nanoparticle fabrication.

## 4 Methods

### 4.1 Beam shaping

A Ti:sapphire laser regenerative amplifier system (Spectra Physics) provided a fundamental Gaussian mode with a central wavelength of 800 nm and pulse duration of 35 fs. The beam diameter (1/*e* ^2^) of the laser is 10 mm. Phase modulation was fulfilled using liquid crystal on a liquid crystal on silicon SLM (Holoeye Pluto). The size of the liquid crystal screen is 15.36 mm × 8.64 mm consisting of 1,920 × 1,080 pixels. The incident beam is carefully centered on the phase pattern displayed on the SLM. A 4*f* system, which consisted of two identical planoconvex lenses with a focusing length of 200 mm, was used to transmit the laser from the SLM to the objective lens without distortion. An Olympus objective (20×, *NA* = 0.45, Entrance Pupil Diameter = 5.6 mm) was used to converge the laser beam. The hollow focusing spot formed at the focusing plane.

### 4.2 Beam characterization

The beam shape at the focal plane of the objective lens cannot be measured easily due to its small size; therefore, the beam shape at the focal plane of the first lens of the 4*f* system is measured by a beam visualizer after strong attenuation of the incident pulse energy. Theoretically, the captured beam shape is a magnification of the focused beam.

Simulation: The complex amplitude representation of the shaped light field incident upon the entrance pupil of the objective lens is articulated in terms of both amplitude and phase:

(3) $$\begin{array}{ll} E_{0}\left(x,y\right) & =A_{0}\exp\left[-\frac{x^{2}+y^{2}}{w^{2}}\right]\exp\left[i\varphi \left(x,y\right)\right]\\ & \;\left(0<\sqrt{x^{2}+y^{2}}<R\right) \end{array}$$

where *A*_0_ is oscillation constant, *w* is waist radius of the laser, $\varphi \left(x,y\right)$ is the additional phase value of the laser after phase shaping, and *R* is the radius of shaping light field.

The shaped optical field, upon being focused by the objective lens, will also generate a lens phase $\varphi_{\mathrm{lens}}\left(x,y\right)$ . Consequently, the complex amplitude representation of the shaped optical field is as follows:

(4) $$\begin{array}{ll} E_{1}\left(x,y\right) & =E_{0}\left(x,y\right)\varphi_{\mathrm{lens}}\left(x,y\right)\\ & =E_{0}\left(x,y\right)\exp\left[-i\frac{k}{2f}\left(x^{2}+y^{2}\right)\right] \end{array}$$

where *f* is the focal length of the lens, *k* = 2π/*λ* (*λ* is the wavelength of laser) is the wave number of incident light. The propagation of the shaped optical field from the objective lens to the focal plane, following its focusing, adheres to the conditions stipulated by the Fresnel approximation:

(5) $$z^{3}\gg \frac{\pi }{4\lambda }{\left[{\left(x_{2}-x_{1}\right)}^{2}+{\left(y_{2}-y_{1}\right)}^{2}\right]}^{2}$$

Therefore, the distribution of diffracted light field can be calculated by Fresnel diffraction integral formula:

(6) $$\begin{array}{ll} E_{2}\left(x_{2},y_{2}\right) & =\frac{\exp\left(ikz\right)}{i\lambda z}\cdot \iint E_{1}\left(x_{1},y_{1}\right)\\ & \;\times \exp\left\{\frac{ik}{2z}\left[{\left(x_{2}-x_{1}\right)}^{2}+{\left(y_{2}-y_{1}\right)}^{2}\right]\right\}dx_{1}y_{1} \end{array}$$

where *z* is the propagation distance of the shaping light field, and the light field distribution of the focal plane can be obtained when *z* = *f*.

### 4.3 Material preparation

Standard polished silica substrates (10 × 10 × 0.5 mm) were under ultrasonic treatment for 30 min and then oxidized in a boiled solution of concentrated sulfuric acid hydrogen peroxide for 10 min. A Cr adhesion layer and Au or Si thin films were deposited sequentially on silica substrate using an electron beam evaporator (Explorer Coating System, Denton Vacuum).

### 4.4 Sample characterization

Scanning electron microscopy (SEM; XL30S-FEG, FEI, Inc.) was used to observe the top view and morphology of the samples. Gold film is sputtered on the surface of the Si film before the SEM measurement.

## Supplementary Material

Supplementary Material Details
