# Supplementary material for: Continuously adjustable hollow beam for ultrafast laser fabrication of size-controllable nanoparticles
Source: Nanophotonics. 2025 Mar 27;14(9):1345–53. doi: 10.1515/nanoph-2024-0690 (PMC12038574; doi:10.1515/nanoph-2024-0690)
Supplement: Supplementary file 1 — Supplementary Material Details [file j_nanoph-2024-0690_suppl_001.docx]

**Supporting Information**

**Continuously adjustable hollow beam for ultrafast laser fabrication of size-controllable nanoparticles**

Zhi Wang^1^, Peng Yi^1^, Andong Wang*^1^, Taoyong Li^1^, Wentao Chen^1^, Xiaolin Qi^1^, and Xiaowei Li^1,2^

*^1^Laser Micro/Nano Fabrication Laboratory, School of Mechanical Engineering, Beijing Institute of Technology, Beijing 100081, China*

*^2^Yangtze Delta Region Academy of Beijing Institute of Technology, Jiaxing 314019, PR China*

**Keywords:** Spatial shaping; Femtosecond laser; Controllably sized nanopar-ticles; Hollow beam.

The vortex beam, characterized by an azimuth phase factor exp (ilθ), possesses orbital angular momentum per photon Lℏ, where L can take any integer value and is referred to as the topological charge. Unlike conventional Gaussian beams, vortex beams exhibit a phase singularity at their center, resulting in a toroidal intensity distribution as depicted in Fig. S1. The helical wavefront of the vortex beam shown in Fig. S1 depends on the topological charge L and determines the number of wound helices and dominant hand.


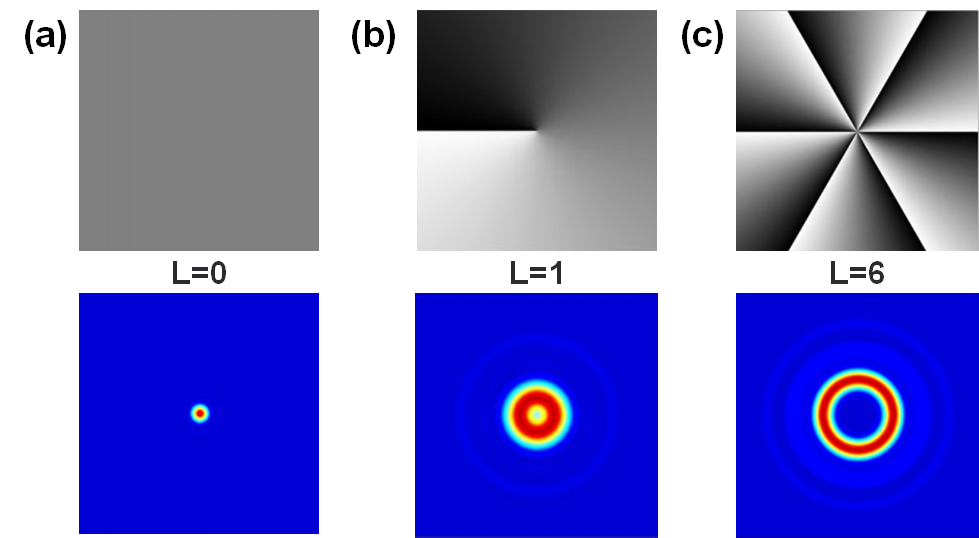


**Figure S1** Phase distribution and corresponding intensity distribution of a vortex beam with topological charge L=0(a),1(b),6(c)

The two beams of light—zeroth order and first order (or first negative order)—generated by off-axis illumination facilitate the imaging of spatial periods p within the range of 0.5λ /NA to λ /NA, significantly enhancing both image contrast and exposure latitude (EL) of the focused spot.

Another advantage of off-axis irradiation is the significant enhancement in Depth of Focus (DOF). In the traditional irradiation method, the incident light is perpendicular to the mask plate plane, resulting in three converging beams in space. It should be noted that at the focal point, the phase of level 0 matches that of level +1 and -1. However, when defocus occurs due to varying distances traveled by ±1 diffracted light compared to coaxial 0-level diffracted light, there comes a certain degree where the phases of level 0 and levels +1/-1 become opposite. This limitation restricts focusing depth. On the other hand, with inclined incident light within a spatial period p ranging from 0.5λ /NA to λ /NA, only two diffraction orders can enter the pupil stage-wise. By precisely adjusting incidence angle such that it equals relative vertical optical axis angle between first-order diffractive light and zero-order diffractive light, both beams have equal phase at any point on optical axis or infinite focal depth even at different defocusing positions can be achieved. However, complete coherence cannot be attained as partial coherence with certain size leads to
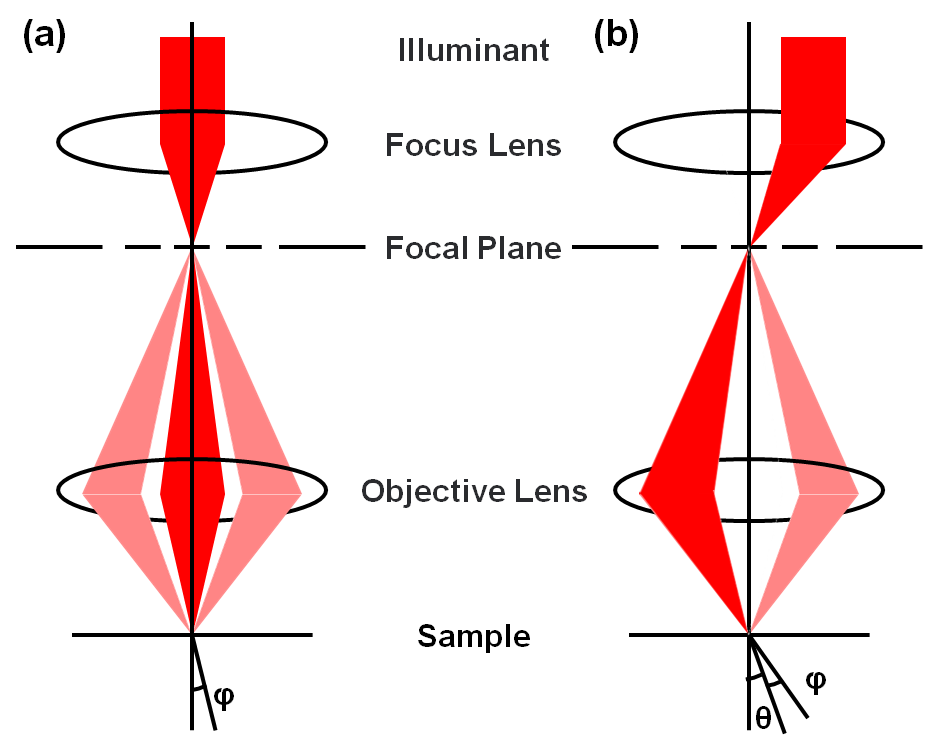
rapid reduction in focal depth towards normal state.

**Figure S2** Normal (a) and Oblique (b) Radiation Focusing Pattern Diagrams


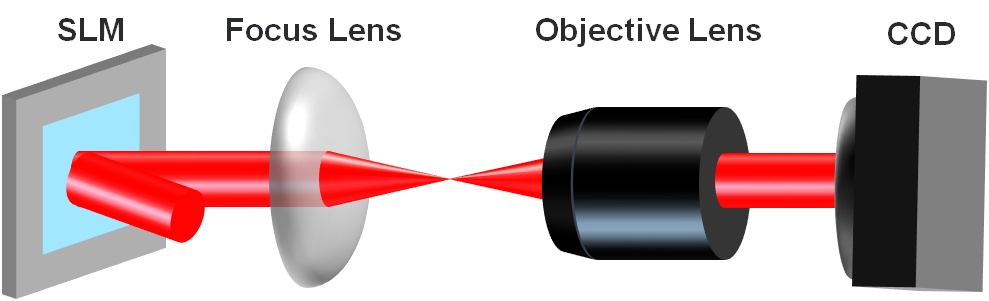
In order to investigate the actual distribution of focusing intensity for hollow beams, an experimental optical system was constructed as depicted in Fig. S3 to evaluate the shaping capability of the hollow beam generation scheme. The optical setup comprises a supercontinuum laser capable of emitting light at any wavelength within the visible spectrum. Following reshaping by a spatial light modulator (SLM), the diffracted XY section is transmitted through a telescope system consisting of an objective lens (50×/NA = 0.6) and a lens with a focal length of 100 mm. Ultimately, the signal is captured by a color CCD.

**Figure S3** Schematic diagram of hollow beam shaping ability test

As the value of d decreases from 1500 μm, the intensity of the π phase beam progressively increases, while that of the 0 phase beam correspondingly diminishes. When d falls below 1300 μm, the intensity of the Π phase beam surpasses that of the 0 phase beam, leading to a π phase focal point within the region of reduced central intensity. This phenomenon results in an energy hotspot, which subsequently causes secondary damage at the center during processing, thereby impacting the effective
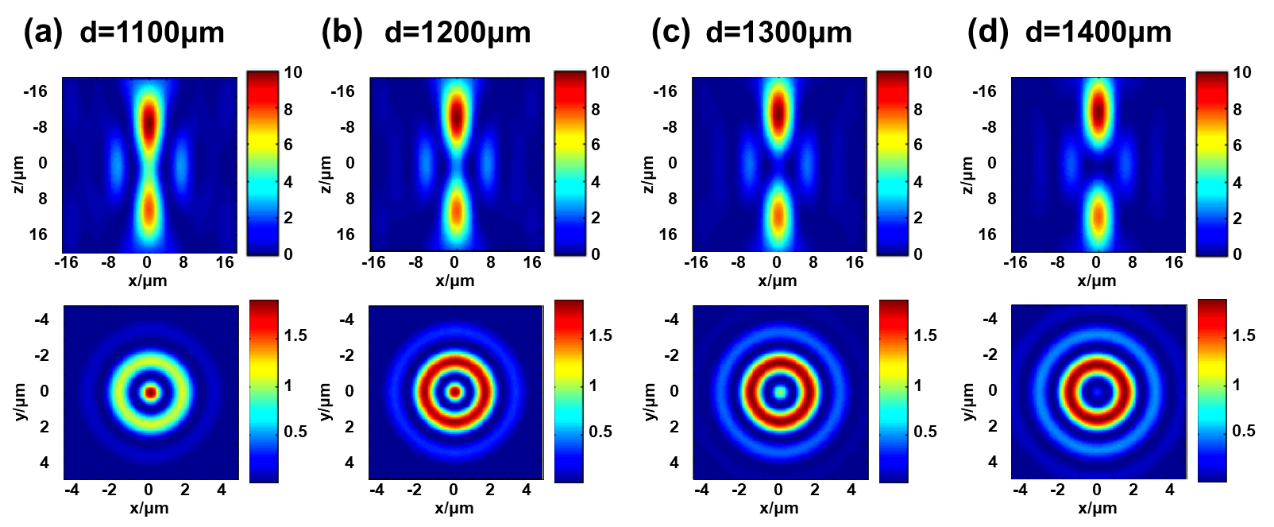
preparation of nanoparticles.

**Figure S4** Simulated axial and lateral intensity distributions of the hollow beam with central diameters of 1100 μm, 1200 μm, 1300 μm, and 1400 μm in MATLAB


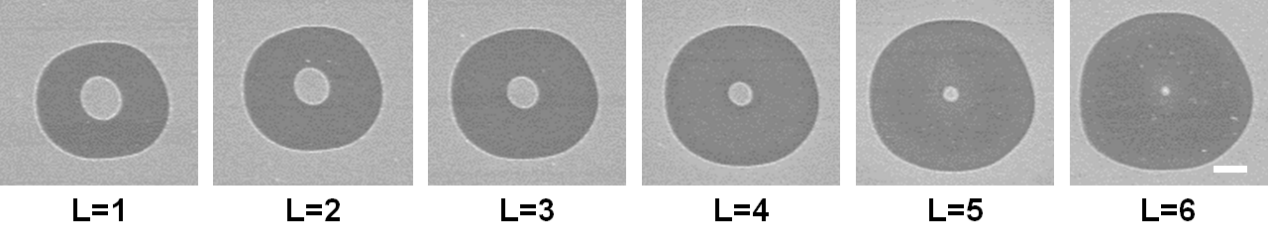
The formation of vortex beams, influenced by the vortex phase, exhibits a significant escalation in side lobe burnout effects and substantial material degradation as the helical degree (L) of the vortex beam varies, while concurrently facilitating a reduction in nanoparticle size.

**Figure S5** SEM characterization results of vortex beams with different helicity (Scale Bar: 2μm)

To further investigate the extent of damage to the substrate caused by nanoparticle preparation, atomic force microscopy (AFM) was employed to characterize the morphology of the nanoparticle preparation region, and surface profiles were extracted for analysis. The results indicate that when the thickness of the gold film is relatively thin (20 nm), the damage threshold of the gold film is lower. Consequently, the volume of gold remelted during femtosecond laser processing is minimal, while the affected area is larger, leading to a shorter cooling time and thus limited damage to the fused silica substrate. Conversely, when the thickness of the gold film is greater (40 nm), the damage threshold increases. This results in a larger volume of gold remelted during femtosecond laser processing, extending the cooling time and consequently causing more significant damage to the fused silica substrate.


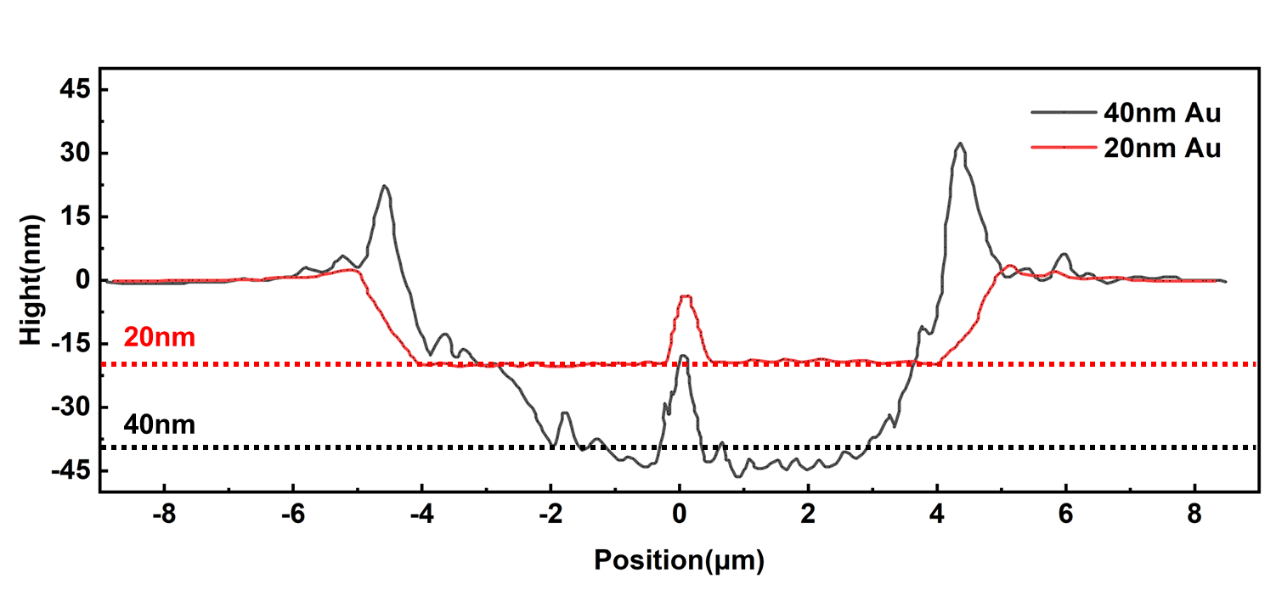


**Figure S6** AFM characterization results of 20nm Au film and 40nm Au film fabricated by Hollow Beam.


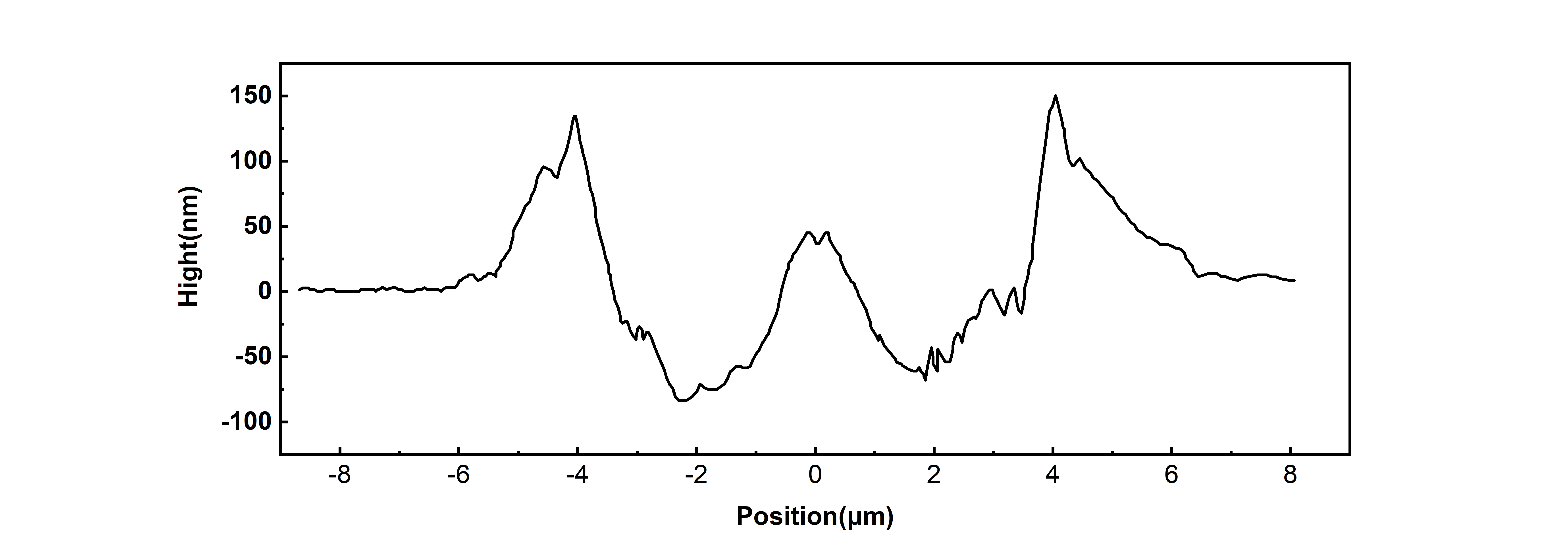


**Figure S7** AFM characterization results of bulk Si fabricated by Hollow Beam.
